# Supplementary material for: Combining vertebrate mitochondrial 12S rRNA gene sequencing and shotgun metagenomic sequencing to investigate the diet of the leopard cat (Prionailurus bengalensis) in Korea
Source: PLoS One. 2023 Jan 31;18(1):e0281245. doi: 10.1371/journal.pone.0281245 (PMC9888693; doi:10.1371/journal.pone.0281245)
Supplement: S1 File — (PDF) [file pone.0281245.s001.pdf]

Supporting Information for:

Combining vertebrate mitochondrial 12S rRNA gene sequencing and shotgun metagenomic sequencing to investigate the diet of the leopard cat (*Prionailurus bengalensis*) in Korea

Short title: DNA sequencing for leopard cat dietary survey

Cheolwoon Woo[1], Priyanka Kumari[1,2], Kyung Yeon Eo [3], Woo-Shin Lee [4], Junpei Kimura [5] and Naomichi Yamamoto[1,2*]

1. Department of Environmental Health Sciences, Graduate School of Public Health, Seoul National University, Seoul 08826, Republic of Korea
2. Institute of Health and Environment, Graduate School of Public Health, Seoul National University, Seoul 08826, Republic of Korea
3. Department of Animal Health and Welfare, College of Healthcare and Biotechnology, Semyung University, Jecheon 27136, Republic of Korea
4. Department of Forest Sciences, College of Agriculture and Life Science, Seoul National University, Seoul 08826, Republic of Korea
5. College of Veterinary Medicine, Seoul National University, Seoul 08826, Republic of Korea

*Correspondence

E-mail: nyamamoto@snu.ac.kr (NY)

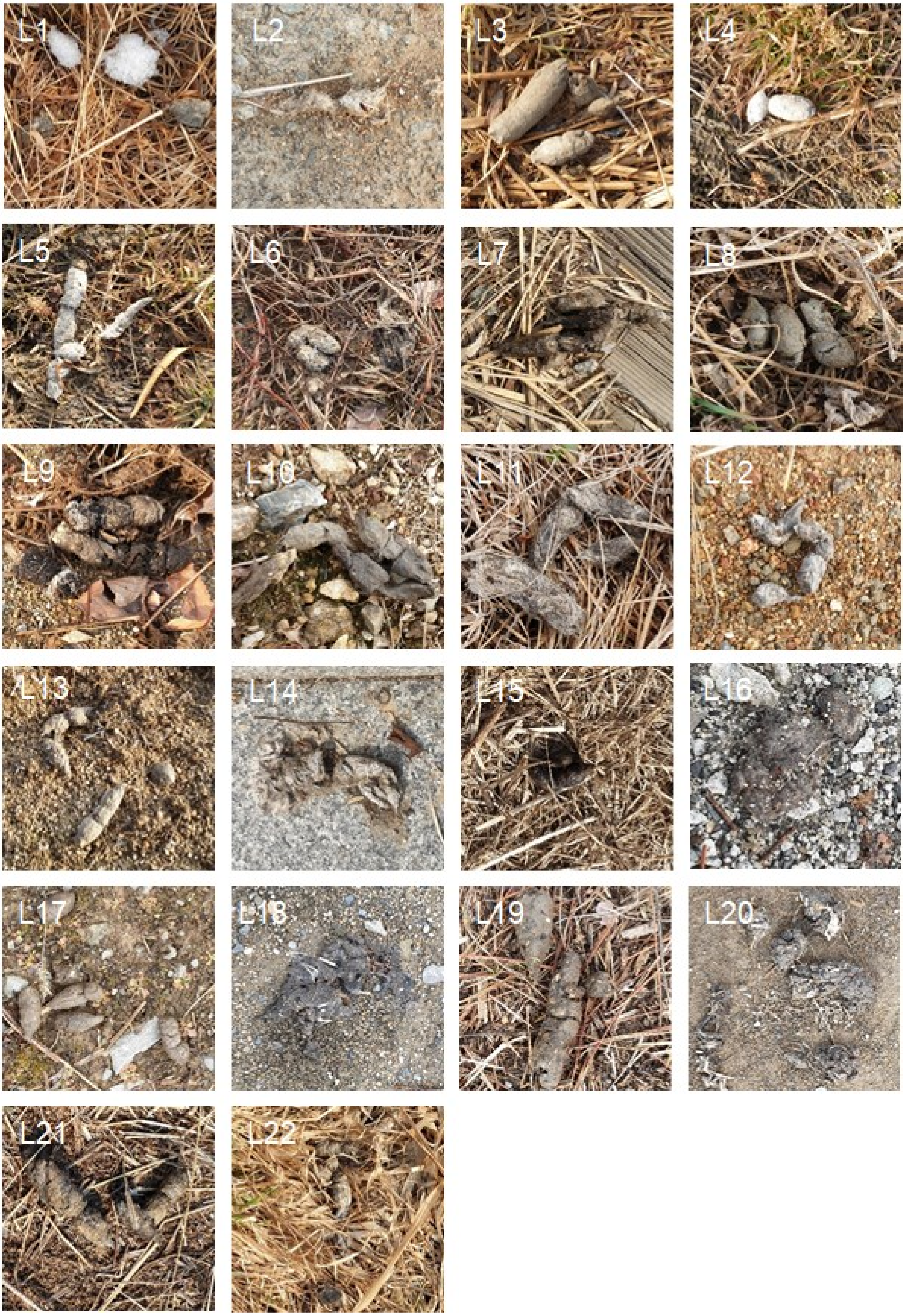

S1 Fig. Fecal samples of leopard cats.

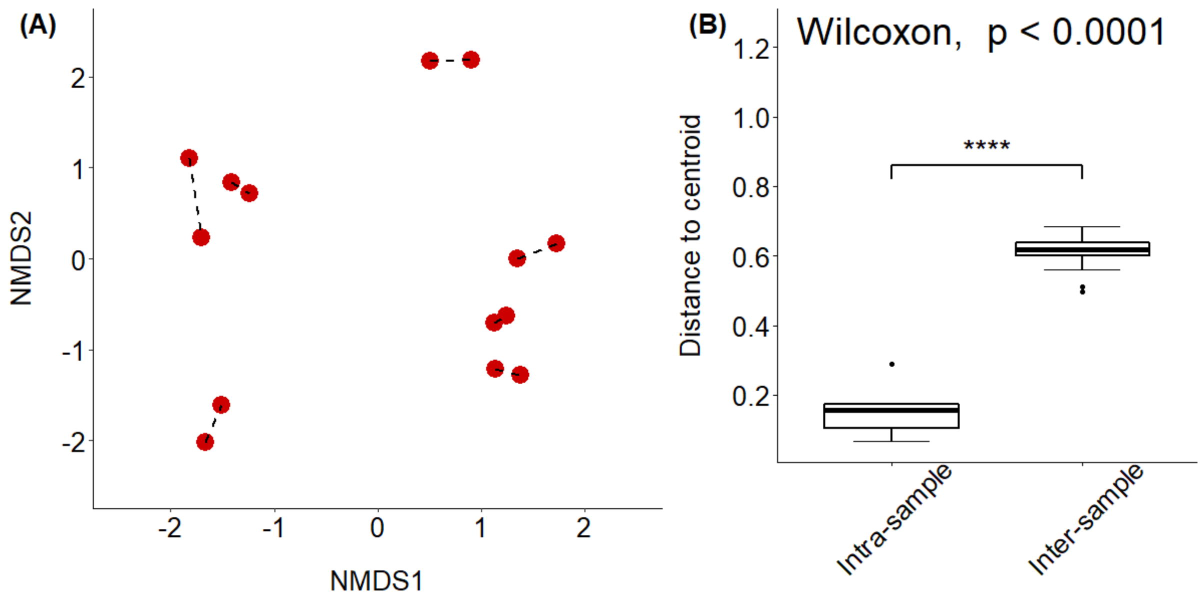

S2 Fig. Reproducibility of vertebrate mitochondrial 12S rRNA gene sequencing. (A) Non-metric multidimensional scaling (NMDS) plot representing the composition of vertebrate prey based on the Bray–Curtis dissimilarity. The data duplicated from the same sample is connected by a line. (B) The comparison between intra- and inter-sample variances of vertebrate prey compositions. The four asterisks (****) represent $p < 0.0001$ by the Wilcoxon rank-sum test.

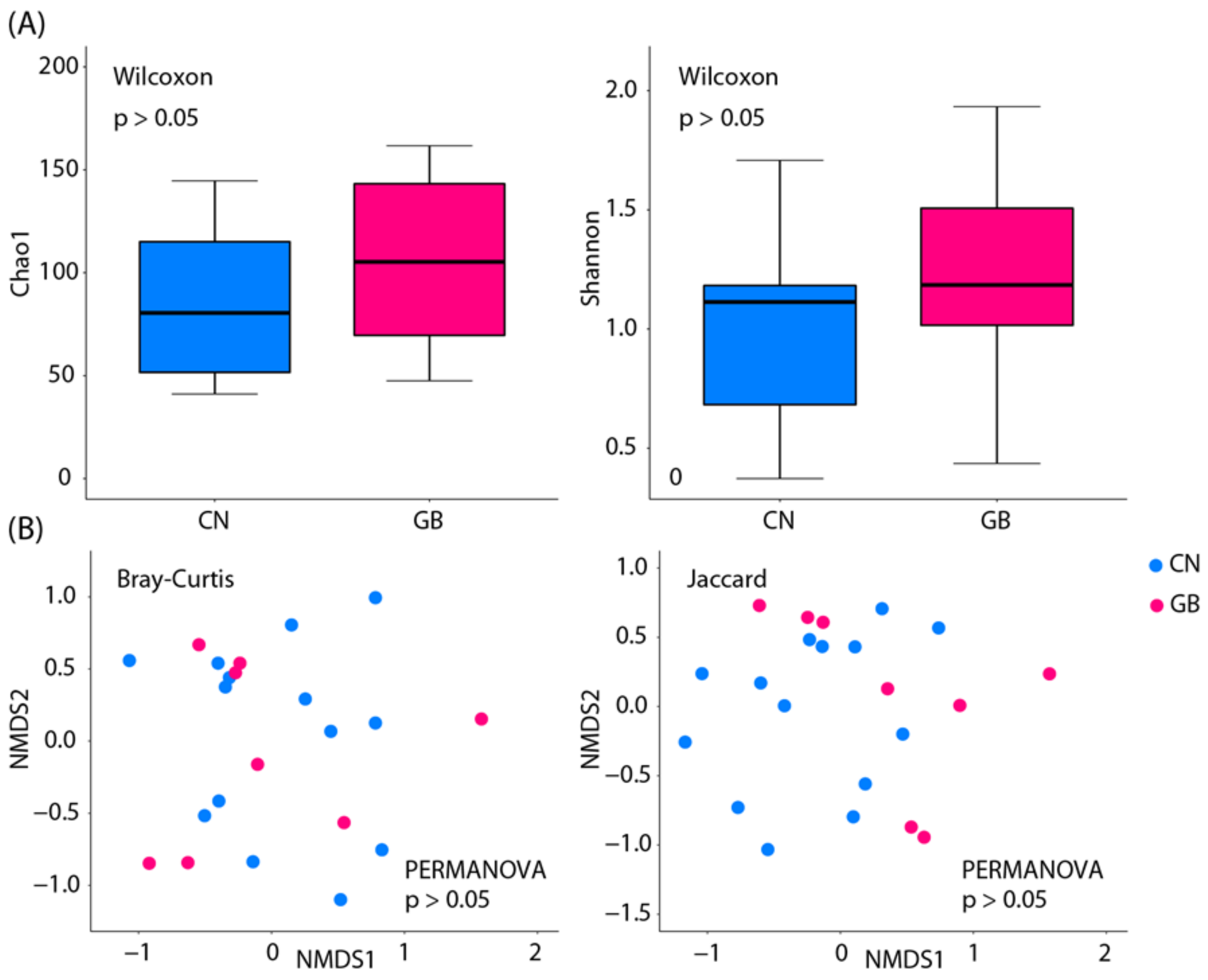

S3 Fig. Spatial comparisons of α and β diversities by vertebrate mitochondrial 12S rRNA gene sequencing. The reads assigned to the family Felidae were excluded from the comparisons, and each library was rarefied to 15,800 reads. (A) Alpha diversity. The Chao1 estimator (community richness) and Shannon index (community diversity) based on OTU-level are shown. (B) Beta diversity. Non-metric multidimensional scaling (NMDS) plots based on the Bray–Curtis dissimilarity (community structure) and Jaccard index (community membership) based on OTU-level are shown. Abbreviation: CN, Chungcheongnam-do (n = 14); and GB, Gyeongsanbuk-do (n = 8).

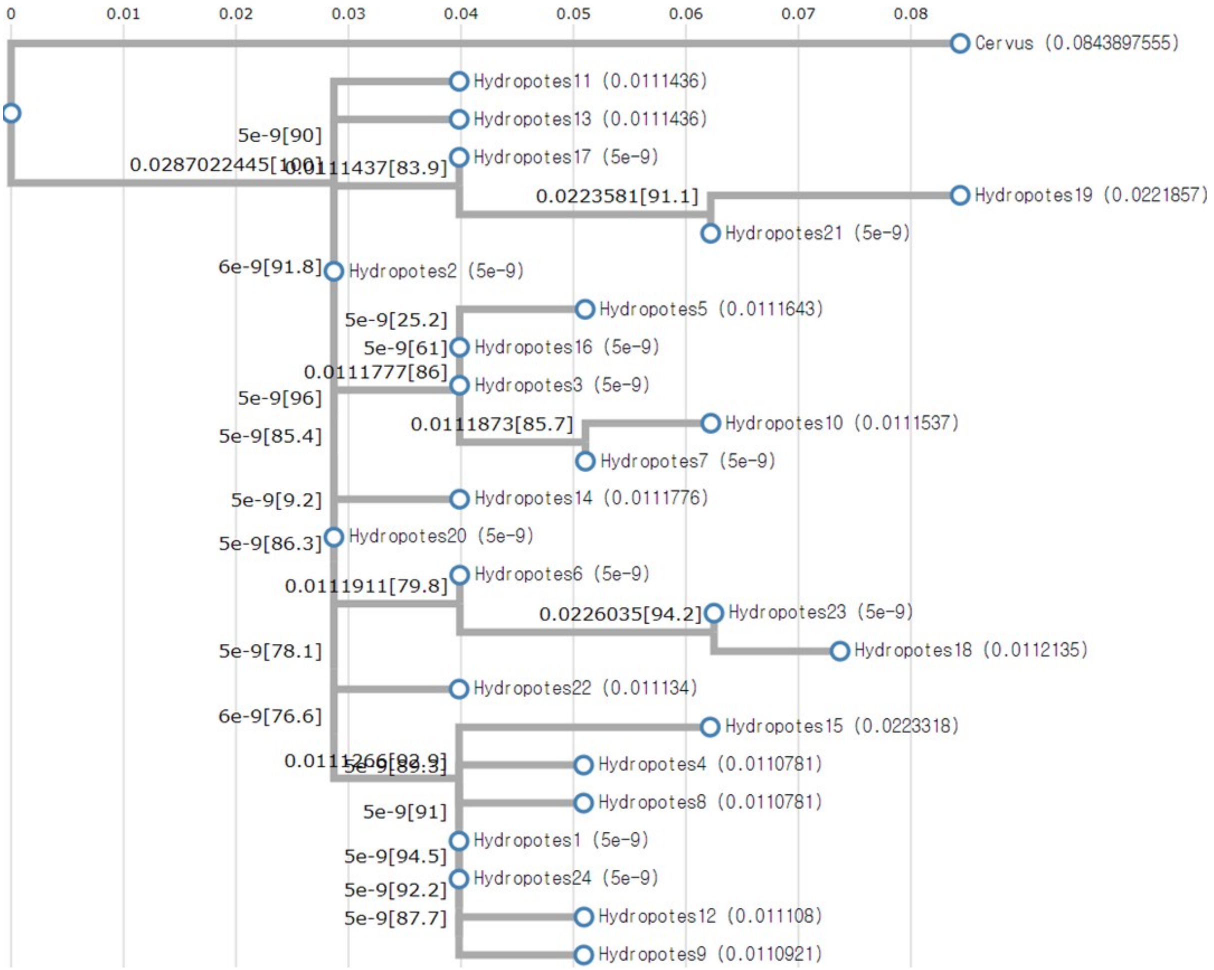

S4 Fig. Phylogenetic tree of vertebrate mitochondrial 12S rRNA gene sequences of OTUs assigned to *Cervus* (n = 1) and *Hydropotes* (n = 24). Clustal W version 2.1 [1] was used with default setting of slow and accurate pairwise alignment method.

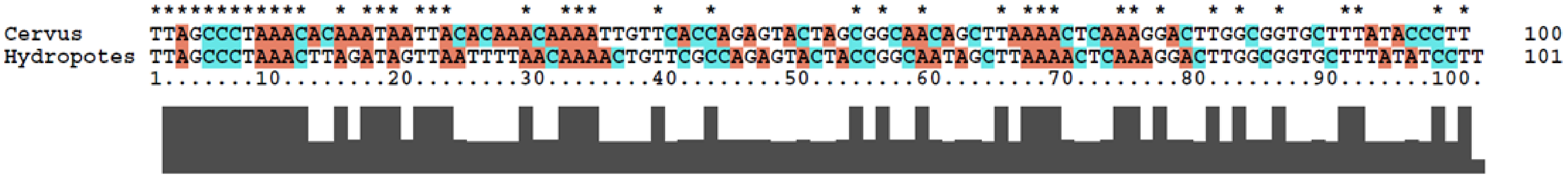

S5 Fig. Alignment of vertebrate mitochondrial 12S rRNA gene sequences of OTUs taxonomically assigned to *Cervus* (n = 1) and *Hydropotes* (n = 24). The centroid sequence was determined against 24 OTUs of *Hydropotes* based on sequence similarity of 0.9, and the determined centroid sequence was used for alignment. Clustal X version 2.1 [1] was used. The gray peak increases as the sequences are similar. A gray peak appears for each nucleotide, and the peak is lowered in case of mismatch.

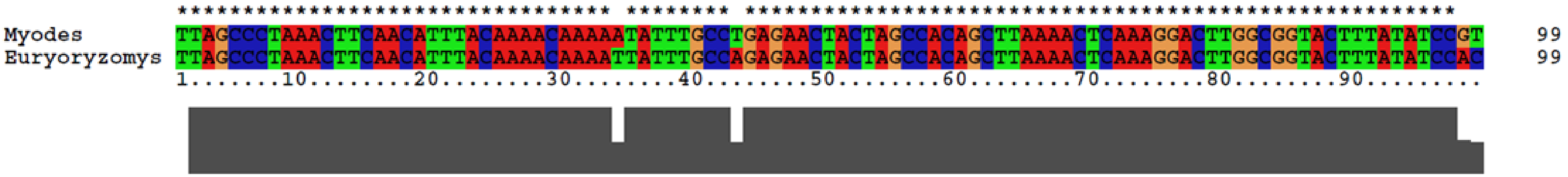

S6 Fig. Alignment of vertebrate mitochondrial 12S rRNA gene sequences of OTUs taxonomically assigned to *Myodes* (n = 1) and *Euryoryzomys* (n = 1). Clustal X version 2.1 [1] was used. The gray peak increases as the sequences are similar. A gray peak appears for each nucleotide, and the peak is lowered in case of mismatch.

S1 Table. Sample metadata.

Sample ID	Sampling date (YYYY/MM/DD)	Latitude	Longitude	City or Gun
L1	2019/02/20	36°36'1.83"N	127°8'2.71"E	Gongju, Chungcheongnam-do
L2	2019/02/20	36°34'32.91"N	127°9'43.39"E	Gongju, Chungcheongnam-do
L3	2019/02/21	36°27'19.5"N	127°9'1.1"E	Gongju, Chungcheongnam-do
L4	2019/02/21	36°27'18.28"N	127°9'2.04"E	Gongju, Chungcheongnam-do
L5	2019/02/21	36°27'17.45"N	127°9'1.79"E	Gongju, Chungcheongnam-do
L6	2019/02/21	36°27'11.21"N	127°8'55.71"E	Gongju, Chungcheongnam-do
L7	2019/02/21	36°30'52.96"N	127°19'41.42"E	Yeongi-gun, Chungcheongnam-do
L8	2019/02/20	36°36'10.53"N	127°7'38.15"E	Gongju, Chungcheongnam-do
L9	2019/02/20	36°36'4.58"N	127°7'54.92"E	Gongju, Chungcheongnam-do
L10	2019/02/20	36°36'4.8"N	127°7'53.11"E	Gongju, Chungcheongnam-do
L11	2019/02/20	36°35'43.81"N	127°8'47.17"E	Gongju, Chungcheongnam-do
L12	2019/02/20	36°35'44.95"N	127°8'46.55"E	Gongju, Chungcheongnam-do
L13	2019/02/21	36°27'17.87"N	127°8'58.7"E	Gongju, Chungcheongnam-do
L14	2019/02/21	36°30'59.4"N	127°19'38.73"E	Yeongi-gun, Chungcheongnam-do
L15	2019/02/05	36°17'55.8"N	128°7'27.12"E	Sangju, Gyeongsangbuk-do
L16	2019/02/20	35°45'13.2"N	128°23'44.52"E	Dalseong-gun, Daegu
L17	2019/02/20	35°44'54"N	128°23'42.72"E	Dalseong-gun, Daegu
L18	2019/02/20	35°44'17.64"N	128°24'3.42"E	Goryeong, Gyeongsangbuk-do
L19	2019/02/20	35°44'18"N	128°24'3.12"E	Goryeong, Gyeongsangbuk-do
L20	2019/02/20	35°44'17.46"N	128°24'3.66"E	Goryeong, Gyeongsangbuk-do
L21	2019/02/20	35°42'52.5"N	128°25'54.36"E	Goryeong, Gyeongsangbuk-do
L22	2019/02/20	35°42'13.8"N	128°23'39.9"E	Goryeong, Gyeongsangbuk-do

S2 Table. Shotgun metagenomic sequencing statistics. The number of sequence reads that are thought to be derived from the diet of the leopard cat is shown [a].

Sample ID	Accession number [b]	No. reads assigned to Chordata [c]	No. reads assigned to Streptophyta	No. reads assigned to Arthropoda	Total
L3	SAMN22418688	18,465	1,364	1,708	21,537
L4	SAMN22418689	20,408	2,187	2,209	24,804
L5	SAMN22418690	5,353	12,235	3,107	20,695
L7	SAMN22418691	12,994	4,430	1,995	19,419
L9	SAMN22418692	3,047	4,611	1,234	8,892
L10	SAMN22418693	7,589	61,264	3,471	72,324
L11	SAMN22418694	6,102	35,360	2,695	44,157
L12	SAMN22418695	5,499	32,524	1,836	39,859
L13	SAMN22418696	26,513	8,840	4,218	39,571
L15	SAMN22418697	17,004	26,046	6,554	49,604
L22	SAMN22418698	2,744	5,347	3,092	11,183
Total		125,718	194,208	32,119	352,045

[a] As the phyla that are thought to be related to the diet of the leopard cat, Chordata, Streptophyta, and Arthropoda were selected.

[b] Raw data has been previously published by our study [2] under the accession number of PRJNA772888 of NCBI.

[c] The reads assigned to the family Felidae to which the leopard cat belongs were excluded because they are likely to be derived from the leopard cat itself (not from the diet).

S3 Table. Vertebrate mitochondrial 12S rRNA gene sequencing statistics.

Sample ID	Accession number	No. sequence reads
L1	SAMN29914670	103,904
L2	SAMN29914671	48,693
L3	SAMN29914672	90,343
L3_dup	SAMN29914672	134,637
L4	SAMN29914673	69,969
L4_dup	SAMN29914673	161,245
L5	SAMN29914674	92,925
L6	SAMN29914675	66,271
L7	SAMN29914676	86,932
L8	SAMN29914677	96,808
L9	SAMN29914678	75,460
L9_dup	SAMN29914678	140,001
L10	SAMN29914679	85,175
L11	SAMN29914680	97,166
L12	SAMN29914681	37,386
L12_dup	SAMN29914681	149,700
L13	SAMN29914682	78,576
L14	SAMN29914683	84,382
L15	SAMN29914684	80,200
L16	SAMN29914685	51,006
L17	SAMN29914686	129,413
L18	SAMN29914687	80,916
L19	SAMN29914688	89,688
L20	SAMN29914689	77,293
L20_dup	SAMN29914689	154,454
L21	SAMN29914690	70,430
L21_dup	SAMN29914690	143,366
L22	SAMN29914691	91,863
L22_dup	SAMN29914691	147,867
Total		2,816,069

S4 Table. Major Streptophyta genera identified by shotgun metagenomic sequencing and their inhabitation status in Korea. Fifteen most abundant genera detected are shown.

Genus	Mean relative abundance (%)	Inhabitation in Korea	Availability of genome
Ricinus	49.8	Confirmed [3]	*Ricinus communis* [4]
Arabidopsis	13.6	Not confirmed	*Arabidopsis thaliana* [5, 6]
Physcomitrella	12.6	Not confirmed	*Physcomitrella patens* [7]
Vitis	5.6	Confirmed [8]	*Vitis vinifera* [9]
Populus	5.3	Confirmed [10]	*Populus trichocarpa* [11]
Oryza	3.6	Confirmed [12]	*Oryza sativa* [13, 14]
Sorghum	3.5	Confirmed [15]	*Sorghum bicolor* [16]
Selaginella	1.2	Confirmed [8]	*Selaginella moellendorffii* [17]
Equisetum	0.4	Confirmed [8]	Not available
Oenothera	0.3	Confirmed [8]	*Oenothera biennis* [18, 19]
Syntrichia	0.3	Confirmed [20]	*Syntrichia caninervis* [21]
Marchantia	0.3	Confirmed [22]	*Marchantia polymorpha* [23]
Chara	0.3	Confirmed [24]	*Chara braunii* [25]
Mesostigma	0.2	Not confirmed	*Mesostigma viride* [26]
Spinacia	0.2	Confirmed [27]	*Spinacia oleracea* [28]

Supporting Reference

1. Larkin MA, Blackshields G, Brown NP, Chenna R, McGettigan PA, McWilliam H, et al. Clustal W and Clustal X version 2.0. Bioinformatics. 2007;23(21):2947–8.
2. Kumari P, Tripathi BM, Dong K, Eo KY, Lee W-S, Kimura J, et al. The host-specific resistome in environmental feces of Eurasian otters (*Lutra lutra*) and leopard cats (*Prionailurus bengalensis*) revealed by metagenomic sequencing. One Health. 2022;14:100385.
3. Lee H-C, CheKar E-K, Lim D-O. The specific plant species and naturalized plants in the area of Naejangsan National Park, Korea. Korean J Environ Ecol. 2011;25(3):267–83.
4. Chan AP, Crabtree J, Zhao Q, Lorenzi H, Orvis J, Puiu D, et al. Draft genome sequence of the oilseed species *Ricinus communis*. Nat Biotechnol. 2010;28(9):951–6.
5. Goodman HM, Ecker JR, Dean C. The genome of *Arabidopsis thaliana*. Proc Natl Acad Sci USA. 1995;92(24):10831–5.
6. The Arabidopsis Genome Initiative. Analysis of the genome sequence of the flowering plant *Arabidopsis thaliana*. Nature. 2000;408(6814):796–815.
7. Rensing SA, Lang D, Zimmer AD, Terry A, Salamov A, Shapiro H, et al. The *Physcomitrella* genome reveals evolutionary insights into the conquest of land by plants. Science. 2008;319(5859):64–9.
8. Lee H-C, Hwang I-C, Lim D-O, Chung C-U. The specific plant species and conservation of Juwangsan National Park, Korea. Korean J Environ Ecol. 2011;25(4):498–515.
9. The French–Italian Public Consortium for Grapevine Genome Characterization. The grapevine genome sequence suggests ancestral hexaploidization in major angiosperm phyla. Nature. 2007;449(7161):463–7.
10. Chung GY, Chang KS, Chung J-M, Choi HJ, Paik W-K, Hyun J-O. A checklist of endemic plants on the Korean Peninsula. Korean J Pl Taxon. 2017;47(3):264–88.
11. Tuskan GA, DiFazio S, Jansson S, Bohlmann J, Grigoriev I, Hellsten U, et al. The genome of black cottonwood, *Populus trichocarpa* (Torr. & Gray). Science. 2006;313(5793):1596–604.
12. Vigueira CC, Qi X, Song B-K, Li L-F, Caicedo AL, Jia Y, et al. Call of the wild rice: *Oryza rufipogon* shapes weedy rice evolution in Southeast Asia. Evol Appl. 2019;12(1):93–104.
13. Goff SA, Ricke D, Lan T-H, Presting G, Wang R, Dunn M, et al. A draft sequence of the rice genome (*Oryza sativa* L. ssp. *japonica*). Science. 2002;296(5565):92–100.
14. Yu J, Hu S, Wang J, Wong GK-S, Li S, Liu B, et al. A draft sequence of the rice genome (*Oryza sativa* L. ssp. *indica*). Science. 2002;296(5565):79–92.
15. Jun HJ, Kang TG, Choi DK, Kim SH. Sorghum harvesting using a head-feeding type rice combine. J Biosyst Eng. 2018;43(4):296–302.
16. Paterson AH, Bowers JE, Bruggmann R, Dubchak I, Grimwood J, Gundlach H, et al. The Sorghum bicolor genome and the diversification of grasses. Nature. 2009;457(7229):551–6.
17. Banks JA, Nishiyama T, Hasebe M, Bowman JL, Gribskov M, dePamphilis C, et al. The *Selaginella* genome identifies genetic changes associated with the evolution of vascular plants. Science. 2011;332(6032):960–3.
18. Greiner S, Wang X, Rauwolf U, Silber MV, Mayer K, Meurer J, et al. The complete nucleotide sequences of the five genetically distinct plastid genomes of *Oenothera*, subsection *Oenothera*: I. sequence evaluation and plastome evolution. Nucleic Acids Res. 2008;36(7):2366–78.
19. Greiner S, Wang X, Herrmann RG, Rauwolf U, Mayer K, Haberer G, et al. The complete nucleotide sequences of the 5 genetically distinct plastid genomes of *Oenothera*, subsection *Oenothera*: II. A microevolutionary view using bioinformatics and formal genetic data. Mol Biol Evol. 2008;25(9):2019–30.
20. Kim W, Higuchi M, Yamaguchi T, Sato T, Inoue Y. New and noteworthy records of the moss flora of Korea. Korean J Pl Taxon. 2020;50(4):419–26.
21. Silva AT, Gao B, Fisher KM, Mishler BD, Ekwealor JTB, Stark LR, et al. To dry perchance to live: Insights from the genome of the desiccation-tolerant biocrust moss *Syntrichia caninervis*. Plant J. 2021;105(5):1339–56.
22. Yim E-Y, Choi B-K, Hyun H-J. Floristic study of bryophytes in Hangyeong Gotjawal (Cheongsu-ri), Jejudo Island. Korean J Pl Taxon. 2019;49(3):215–23.

23. Bowman JL, Kohchi T, Yamato KT, Jenkins J, Shu S, Ishizaki K, et al. Insights into land plant evolution garnered from the *Marchantia polymorpha* genome. Cell. 2017;171(2):287–304.
24. Choi KC, Kim YH. Taxonomic study on the charophytes in Korea I. *Chara* and *Lamprothamnium*. Algae. 1997;12(3):177–206.
25. Nishiyama T, Sakayama H, de Vries J, Buschmann H, Saint-Marcoux D, Ullrich KK, et al. The *Chara* genome: Secondary complexity and implications for plant terrestrialization. Cell. 2018;174(2):448–64.
26. Lemieux C, Otis C, Turmel M. Ancestral chloroplast genome in *Mesostigma viride* reveals an early branch of green plant evolution. Nature. 2000;403(6770):649–52.
27. Yoon Y-E, Kuppusamy S, Kim SY, Kim JH, Lee YB. Free amino acid composition of Korean Spinach (*Spinacia oleracea*) cultivars as influenced by different harvesting time. Korean J Environ Agric. 2016;35(2):104–10.
28. Xu C, Jiao C, Sun H, Cai X, Wang X, Ge C, et al. Draft genome of spinach and transcriptome diversity of 120 *Spinacia* accessions. Nat Commun. 2017;8(1):15275.
