# Supplementary material for: Combining vertebrate mitochondrial 12S rRNA gene sequencing and shotgun metagenomic sequencing to investigate the diet of the leopard cat (Prionailurus bengalensis) in Korea
Source: PLoS One. 2023 Jan 31;18(1):e0281245. doi: 10.1371/journal.pone.0281245 (PMC9888693; doi:10.1371/journal.pone.0281245)
Supplement: Attachment — Submitted filename: Response.docx [file pone.0281245.s003.docx]

 **TO:**	Prof. Michael Schubert, Academic Editor of *PLoS ONE*

**FROM:**	Cheolwoon Woo, Priyanka Kumari, Kyung Yeon Eo, Woo-Shin Lee, Junpei Kimura, and Naomichi Yamamoto, authors of “Combining vertebrate mitochondrial 12S rRNA gene sequencing and shotgun metagenomic sequencing to investigate the diet of the leopard cat (*Prionailurus bengalensis*) in Korea”

**RE:**	Response to reviewer comments

**DATE:**	January 9, 2023

Please find our responses to the reviewers’ comments below. The parts changed are marked in the revised manuscript, and the line numbers in our responses refer to the revised manuscript with track changes.

**Reviewer #1:**

**1. The authors have addressed my previous comments, and the current form of the manuscript is suitable for publication.**

**RESPONSE:** Thank you for your interest in reviewing our submitted paper and the paper we have revised to reflect your opinion. We were able to study a lot while studying your comments and found that the revised paper was of better quality. Once again, thank you for your interest in our paper and for your time.

**Reviewer #2:**

**1. The authors have addressed the majority of my comments satisfactorily.**

**RESPONSE:** Thank you for your interest in reviewing our submitted paper and the paper we have revised to reflect your opinion. We were able to study a lot while studying your comments and found that the revised paper was of better quality. Once again, thank you for your interest in our paper and for your time.

**2. However, I believe they misunderstood my comment #6 (pasted below):

L244-249: It would be interesting to know whether there was any correlation between the 12S results and metagenomic results within each individual, i.e. using a mantel test or similar multivariate test.

The authors' response:
Thank you for your suggestion. If we understand your suggestion correctly, your suggestion is to examine the leopard cat individual from which each fecal sample was derived, and then compare the results of vertebrate mitochondrial 12S rRNA gene sequencing and shotgun metagenomic sequencing within each leopard cat individual. As answered in your third question, we tried to determine whether the samples used for shotgun metagenomic sequencing were derived from the same individual. However, due to limited recovery and shorter size of the shotgun metagenomic sequencing reads, it is not feasible to assemble these reads into larger genomic contigs to perform such analysis.

My clarification:
Conducting a mantel (or similar) test should not require the metagenomic sequencing results to be assembled into longer contigs. On the contrary, these tests are often used to compare distance matrices composed of different data types. They could be used to determine whether the results from the eleven cats analyzed by both 12S and metagenomic sequencing result in similar euclidean distances. I recommend the authors look at how Li et al. 2017 (doi: 10.3389/fmicb.2017.00454) used mantel tests to determine correlations between different distances including host phylogenetic distance, gut microbiota, and metabolites. A similar approach could be used to determine whether 12S and metagenomic sequencing results were correlated within each individual.**

**RESPONSE:** Thank you for your clarification. We are sorry that we misunderstood your question. According to your suggestion, the Mantel test was conducted.

To proceed with the Mantel test, first, distance matrices were obtained at the class level from 12S and metagenomic sequencing data. These distance matrices, based on Bray–Curtis dissimilarity, are the same ones we used to create Fig 3C. Then, the Mantel test was conducted based on Spearman’s rank correlation analysis, in order to evaluate the correlation between distance matrices of 12S and metagenomic sequencing. As a result of the Mantel test, no correlation was found between the two sequencing data at the class level. This seems to be caused by the incompleteness of the reference database used for metagenomic sequencing, as discussed in our paper. In addition, the performance of the Mantel test and its results have been added to the paper as follows.

“Additionally, the Mantel test was performed based on the Bray–Curtis dissimilarity with 10,000 permutations, in order to evaluate the Spearman’s rank correlation between the distance matrices of shotgun metagenomic sequencing and vertebrate mitochondrial 12S rRNA gene sequencing.” Lines 194-197

“No correlation was found between the distance matrices of shotgun metagenomic sequencing and vertebrate mitochondrial 12S rRNA gene sequencing at the class level (*r* = −0.05, *p* > 0.05; Mantel test).” Lines 272-274
